# Real-Time Closed-Loop Detection Method of vSLAM Based on a Dynamic Siamese Network

**DOI:** 10.3390/s21227612

**Published:** 2021-11-16

**Authors:** Quande Yuan, Zhenming Zhang, Yuzhen Pi, Lei Kou, Fangfang Zhang

**Affiliations:** 1 School of Computer Technology and Engineering, Changchun Institute of Technology, Changchun 130012, China; yuanqd@airlab.ac.cn; 2 National Local Joint Engineering Research Center for Smart Distribution, Grid Measurement and Control with Safety Operation Technology, Changchun Institute of Technology, Changchun 130012, China; 3 School of Electrical Engineering, Northeast Electric Power University, Jilin 132011, China; 15143289522@163.com; 4 School of Electrical Engineering and Information Technology, Changchun Institute of Technology, Changchun 130012, China; 5 Institute of Oceanographic Instrumentation, Qilu University of Technology (Shandong Academy of Sciences), Qingdao 266075, China; koulei1991@hotmail.com; 6 School of Electrical Engineering and Automation, Qilu University of Technology (Shandong Academy of Sciences), Jinan 250353, China; zhff4u@qlu.edu.cn

**Keywords:** simultaneous localization and mapping, closed-loop detection, Siamese network, deep learning, elementwise integration strategy

## Abstract

As visual simultaneous localization and mapping (vSLAM) is easy disturbed by the changes of camera viewpoint and scene appearance when building a globally consistent map, the robustness and real-time performance of key frame image selections cannot meet the requirements. To solve this problem, a real-time closed-loop detection method based on a dynamic Siamese networks is proposed in this paper. First, a dynamic Siamese network-based fast conversion learning model is constructed to handle the impact of external changes on key frame judgments, and an elementwise convergence strategy is adopted to ensure the accurate positioning of key frames in the closed-loop judgment process. Second, a joint training strategy is designed to ensure the model parameters can be learned offline in parallel from tagged video sequences, which can effectively improve the speed of closed-loop detection. Finally, the proposed method is applied experimentally to three typical closed-loop detection scenario datasets and the experimental results demonstrate the effectiveness and robustness of the proposed method under the interference of complex scenes.

## 1. Introduction

SLAM (simultaneous localization and mapping) is one of the core problems in mobile robot research [1,2], which can incrementally build a continuous map of the surrounding environment in an unknown environment. Compared with laser sensors, vision sensors have significant advantages such as perception capability, flexibility, and cost effectiveness. At the same time, with the rapid development of computer vision technology and the successful integration with vSLAM, vSLAM based on deep learning has become a research hotspot in the field [3,4,5].

SLAM is not a one-way process, and the process of moving and building a map in a region will inevitably go through locations that have been visited before. Closed-loop detection [6,7] judges whether the mobile robot has returned to the visited position and obtains an accurate position of the robot through the constructed map, whose core goal is to select accurate key frame images. If the correct closed-loop is detected effectively, the cumulative error of the system can be significantly reduced. Otherwise, the system back-end optimization algorithm will converge to a completely incorrect value [8]. Therefore, accurate and stable closed-loop detection is very important for a vSLAM system.

Since 2015, researchers have tried to apply deep learning to SLAM closed-loop detection [9,10]. Luo et al. [11,12] adopted a stacked AutoEncoder for deep image feature extraction and employed a similarity matrix to detect the closed-loop. Compared with the traditional fab-map (fast appearance-based mapping) method on the public dataset, it has gained better results. Shamwell et al. [13,14] describe an unsupervised deep neural network method that fuses RGB-D images with inertial measurements for an absolute trajectory estimation, solving the localization problem with an online error correction. Lan et al. [15] adopted trained convolutional neural networks (CNNs) to extract the features of the image and combined them with an adaptive similarity matrix to improve the robustness of the loop closure detection to a certain extent. Cascianelli et al. [16,17] employed an edge boxes segmentation algorithm to divide the image and adopted a trained deep network to extract the features in image block to realize a semi-semantic closed-loop detection. Wang et al. [18] proposed an unsupervised learning method for visual tracking where the tracker was able to forward localize the target object in consecutive frames and retrace back to its initial position in the first frame. Ma et al. [19,20] adopted a place convolutional neural network (PlaceCNN) to extract image features for loop closure detection and achieved good detection results in a complex illumination.

**Figure 1:**
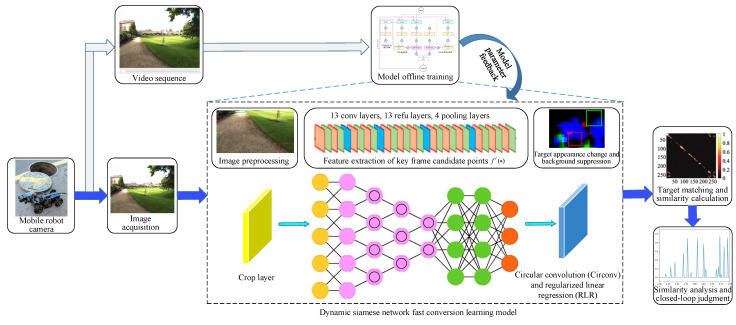
Execution flow of the real-time closed-loop detection method.

The above studies are successful applications in vSLAM loop closure detection from different perspectives such as deep features, semantic information, target detection, and local scenes, which have improved the accuracy and robustness of loop closure detection to varying degrees. However, the loop closure detection results are still unsatisfactory in the face of a complex scene (strong light and jitters) [21]. Most existing high-dimensional feature extraction methods struggle to meet the real-time needs of loop closure detection. To solve the above problems, a real-time closed-loop detection method of vSLAM based on a dynamic Siamese network is proposed in this paper. The main flow of this method is shown in Figure 1. The effectiveness and real-time performance of the method is validated by a typical dataset of closed-loop detection scenarios.

As illustrated in Figure 1, a mobile robot achieves the image information acquisition of the experienced environment during simultaneous positioning and map construction. We construct a fast conversion learning model based on a dynamic Siamese network, which ensures the online learning result of the changing target and background suppression in the environment image captured by the visual sensor. The crop layer implements the pre-processing work on the image information. A combined neural network is used for the feature extraction of key frame candidates. Circular convolution (CirConv) and regularized linear regression (RLR) network layers work on the target appearance change and background suppression, respectively. The closed-loop judgment task is achieved by target matching and a similarity calculation. Through the closed-loop detection of continuous video sequence training in the motion process of the mobile robot, we ensure the offline learning of all parameters and make full use of the rich spatiotemporal information in the motion process.

The main contributions of this paper are as follows:

- We design a real-time closed-loop detection method of vSLAM based on a dynamic Siamese network. Through a fast conversion learning model, the appearance change and background suppression of continuous key frames can be learned online using the first few frames of the images, which can effectively improve the closed-loop detection speed whilst retaining the online adaptive ability as much as possible.
- An elementwise fusion strategy is proposed to adaptively integrate the multi-layer depth features that truly reflects the complementary role of response mapping in the different layers and helps to obtain a better key frame positioning ability.
- The closed-loop detection training based on the labeled video sequence optimizes the traditional discriminant analysis method based on static image similarity so that the closed-loop detection model can fully consider the rich space–time information in the process of robot motion and then obtain a more accurate closed-loop detection effect.

The remainder of this paper is organized as follows. The detailed discussion of the dynamic Siamese network fast conversion learning model is in Section 2. Section 3 provides the core part of the closed-loop detection training mechanism considering the spatiotemporal correlation. Section 4 presents the experimental comparison results and performance analysis of the proposed method. Finally, the conclusions and future recommendations are contained in Section 5.

## 2. Fast Conversion Learning Model Based on a Dynamic Siamese Network

The high level architecture of the vSLAM real-time closed-loop detection method is given in Figure 1. It can be divided into three core components: (i) a fast conversion learning model based on dynamic Siamese networks; (ii) the target appearance change and background suppression conversion mechanism; (iii) the elementwise fusion strategy. These three different functional neural network layers are centralized and synthesized into a unified architecture, abstractly expressed as described in Section 2.4.

### 2.1. Fast Conversion Learning Model Idea

In this paper, vSLAM real-time closed-loop detection is defined as the joint problem of fast template matching and online transformation learning based on continuous frame information. Therefore, based on the classical Siamese network [22], we added two online updatable components *M* and *N* to the two branches of a static Siamese network to update the depth characteristics of the target template and exploration area in the closed-loop detection process, respectively. Component *M* realizes the transformation of the target appearance change during the robot movement and component *N* highlights the depth features of the key point area of the target template to suppress the interference of irrelevant background features. The optimized static Siamese network is more suitable for the needs of dynamic closed-loop detection, which can be defined as Equation (1):

(1) $$S_{t}^{l}=corr(M_{t-1}^{l}*f^{l}(O_{1}),N_{t-1}^{l}*f^{l}(Z_{t}))$$

where $S_{t}^{l}$ represents the response mapping of the target at the possible position of frame *t*, * represents a cyclic convolution operation that can be solved quickly in the frequency domain without changing the input size, $O_{1}$ represents the key frame target template, and $M_{t-1}^{l}$ represents the target appearance change conversion obtained from the online learning of frame *t* − 1 by considering the time smooth change of the target. $M_{t-1}^{l}$ can promote the similarity between $f^{l}(O_{1})$ and $f^{l}(O_{t-1})$, $Z_{t}$ represents the exploration area, and $N_{t-1}^{l}$ represents the background suppression conversion.

In this paper, a regularized linear regression (RLR) is used to calculate the transformation of target appearance change $M_{t-1}^{l}$ and background suppression conversion $N_{t-1}^{l}$ The tensors *X* and *Y* are defined to solve the optimal linear transformation matrix *T*:

(2) $$T=\underset{\Theta }{\arg\min}{\left\|\Theta *X-Y\right\|}^{2}+\lambda {\left\|\Theta \right\|}^{2}.$$

As the essence of the discrete Fourier transform (DFT) is the transformation of a periodic sequence to a frequency domain [23], we used this property to realize the fast solution of linear transformation matrix *T* in the frequency domain:

(3) $$T=\Gamma^{-1}\left(\frac{\bar{\Gamma }(X)⊙\Gamma (Y)}{\bar{\Gamma }(X)⊙\Gamma (X)+\lambda }\right)$$

where Γ represents DFT, $\Gamma^{-1}$ represents the inverse operation of DFT, and $\bar{\Gamma }$ represents the complex conjugate operation of DFT.

### 2.2. Online Learning of the Target Appearance Change and Background Suppression

This subsection introduces the specific implementation process of the target appearance change and background suppression conversion mechanism in two main parts.

#### 2.2.1. Solution of the Target Appearance Change Transformation

The process of SLAM was carried out in parallel with the spatial physical movement of the mobile robot. High quality closed-loop detection requires the timely update of key frame target templates.

**Figure 2:**
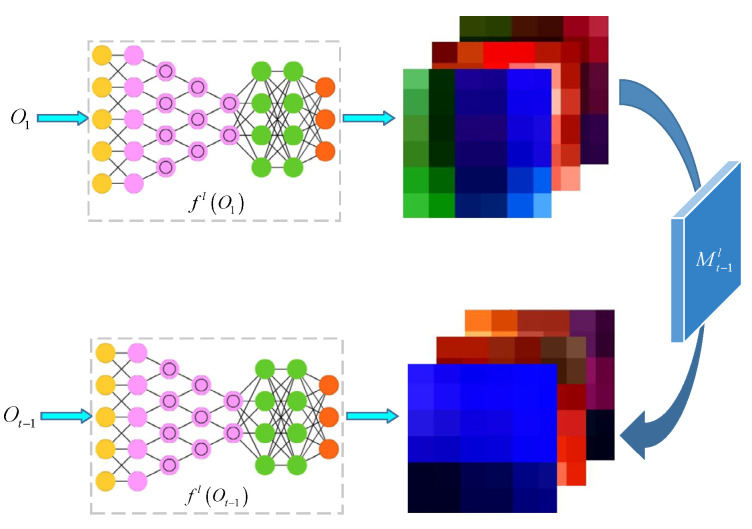
Conversion of the target appearance change.

When in *t* − 1 frame, the target template $O_{t-1}$ of the image at this time can be easily obtained. Instead of simply using $O_{t-1}$ to replace the original target template $O_{1}$, grasp the appearance change from $O_{1}$ to $O_{t-1}$ and apply this change to make $f^{l}(O_{1})$, similar to $f^{l}(O_{t-1})$ according to Equation (1), to reduce the probability of false positive detection caused by complex scene changes such as illuminations or jitters (Figure 2).

Based on Equation (2), the target appearance change transformation a is obtained. The formula is shown in Equation (4):

(4) $$M_{t-1}^{l}=\underset{M}{\arg\min}{\left\|M*f^{l}(O_{1})-f^{t}(O_{t-1})\right\|}^{2}+\lambda_{m}{\left\|M\right\|}^{2}$$

where $\lambda_{m}$ controls the degree of regularization obtained by training the labeled video sequences, which will be described in Section 3. Through Equation (3), Equation (5) is obtained:

(5) $$M_{t-1}^{l}=\Gamma^{-1}\left(\frac{\bar{\Gamma }(f^{l}(O_{1}))⊙\Gamma (f^{l}(O_{t-1}))}{\bar{\Gamma }(f^{l}(O_{1}))⊙\Gamma (f^{l}(O_{t-1}))+\lambda_{m}}\right).$$

#### 2.2.2. Solution of the Background Suppression Transformation

**Figure 3:**
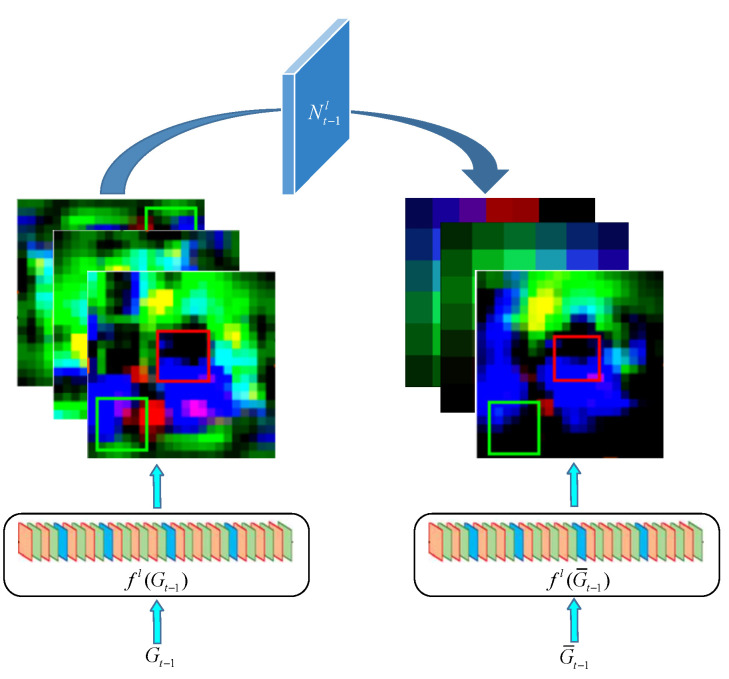
Conversion process of the background suppression.

The goal of closed-loop detection is to accurately obtain the key frame image with the highest similarity to the position visited by the mobile robot so that the whole map can be more accurate. Therefore, reducing the interference of the background to the candidate points in the key frame image selection helps to further improve the closed-loop detection accuracy. Suppose at time t, according to the cumulative learning of the previous *t* − 1 frame, obtain the position of the key candidate points and cut the whole frame image $I_{t-1}$ to the area $G_{t-1}$ centered on the key candidate points. Ensure that the search area $Z_{t-1}$ has the same size. Multiply $G_{t-1}$ by the Gaussian weight mapping [24] to obtain the key candidate points highlighted to suppress the depth characteristics of the background area. Finally, in order to avoid the loss of the original keyframe background depth features, define the background suppression conversion $N_{t-1}^{l}$ to promote the depth features $G_{t-1}$ to be as similar to ${\bar{G}}_{t-1}$ as possible. The background suppression conversion process is shown in Figure 3.

According to Equation (2), the specific formula for solving $N_{t-1}^{l}$ is as follows:

(6) $$N_{t-1}^{l}=\underset{N}{\arg\min}{\left\|N*f^{l}(G_{t-1})-f^{l}({\bar{G}}_{t-1})\right\|}^{2}+\lambda_{n}{\left\|N\right\|}^{2}.$$

Through Equation (3), it can be solved:

(7) $$N_{t-1}^{l}=\Gamma^{-1}\left(\frac{\bar{\Gamma }\left(f^{l}(G_{t-1})\right)⊙\Gamma \left(f^{l}({\bar{G}}_{t-1})\right)}{\Gamma \left(f^{l}(G_{t-1})\right)⊙\Gamma \left(f^{l}(G_{t-1})\right)+\lambda_{n}}\right).$$

By learning the appearance environment change and background suppression of key frames in the process of robot mapping, the dynamic Siamese network is guaranteed to have an online adaptive ability to obtain higher closed-loop detection accuracy and more impressive real-time performance. In addition, the model parameters $\lambda_{m}$ and $\lambda_{n}$ are determined by marking the video sequence learning, which affects the depth feature of key frame candidate points in closed-loop detection rather than the human-designed feature in the traditional way. For example, closed-loop detection methods such as the bag of visual word (BoVW) based method [25] and generalized search tree (GiST) [26], whose image features are artificially designed and are very sensitive to complex scene changes in the environment, affect the success rate of closed-loop detection to a certain extent.

### 2.3. Adaptive Fusion of the Multi-Layer Depth Features

In the process of closed-loop detection, when the key frame candidate points are close to the center of the search area, the deeper features help to eliminate the background interference and the shallower features help to locate the candidate points accurately. When key frame candidate points are located at the periphery of the search area, only deeper features can effectively determine the location of the candidate points. Therefore, in order to obtain a more effective key frame candidate location ability, the proposed dynamic Siamese network adopts an elementwise fusion strategy to realize the complementarity of response mapping at different layers [27]. The response mapping of multi-level features of the deep feature network $\left\{S_{t}^{l}|l\in L\right\}$ is generated by Equation (1) where L represents the number of layers of the entire deep feature network. Set the elementwise weight mapping for the output $S_{t}^{l}\in {Ρ}^{i_{S}\times j_{S}}$ of each layer and let $\sum_{l\in L}\Phi^{l}=1_{i_{S}\times j_{S}}$. The offline learning of $\Phi^{l}$ is described in Section 3. Therefore, the final response mapping can be expressed as follows:

(8) $$S_{t}=\sum_{l\in L}\Phi^{l}⊙S_{t}^{l}$$

where ⊙ represents the elementwise multiplication, which multiplies the output $S_{t}^{l}$ of each layer one by one with the elements in the corresponding weight map $\Phi^{l}$. The adoption of this strategy can bring two advantages:

- The spatial variable integration of deep features realizes the effective integration of the element level.
- Weight mapping $\Phi^{l}$ can be learned offline, replacing the traditional subjective manual setting.

### 2.4. Architecture of the Dynamic Siamese Network

**Figure 4:**
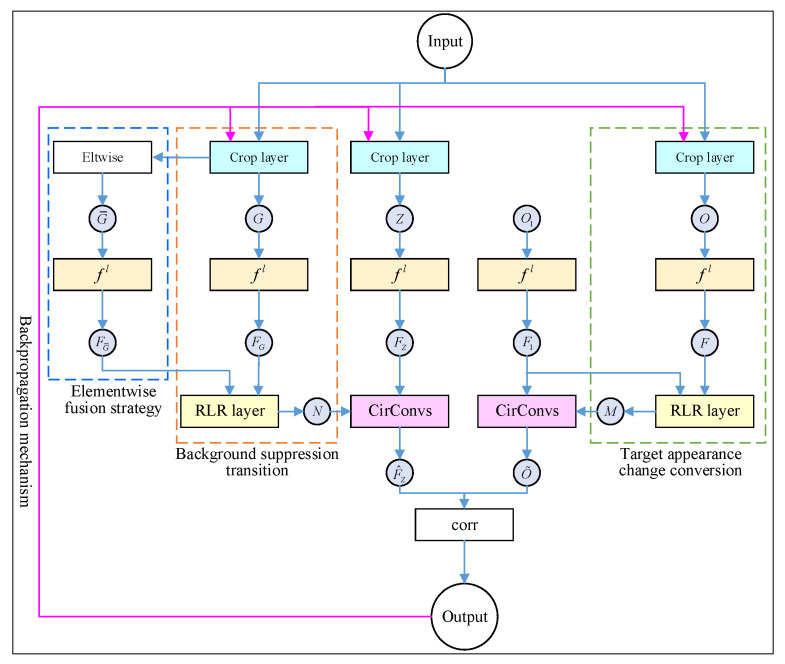
Architecture of the dynamic Siamese network.

Combined with Equations (1), (5), and (7), the dynamic Siamese network was obtained by using the single-layer depth feature. The specific network architecture is shown in Figure 4. The dynamic Siamese network based on the single-layer depth feature was further extended to the multi-layer version of the dynamic Siamese network through the elementwise fusion strategy of Equation (8).

In Figure 4, $f^{l}(\cdot )$ represents the L-th layer depth characteristics of the appropriate CNN model such as AlexNet and VGGNet. On this basis, two new network layers were introduced: CirConv and RLR. The fast conversion and learning ability of Equations (5) and (7), $M_{t-1}^{l}$ and $N_{t-1}^{l}$, were integrated into the unified network system where $M_{t-1}^{l}$ implemented the depth feature update of $O_{1}$ and $N_{t-1}^{l}$ aimed to highlight the depth features of the target neighborhood and reduce the interference of irrelevant background features. A detailed description is given in Section 2.1. To make the closed-loop detection model constructed based on the dynamic Siamese network in this paper to be trained directly on the marked video sequence not just on the image pair, a crop layer was added to the network system to obtain $Z_{t}$, $O_{t-1}$, $G_{t-1}$, and ${\bar{G}}_{t-1}$ according to the response map $S_{t-1}^{l}$, which made the training loss effectively back-propagate from the last frame to the first frame. The specific discussion is in Section 3. In addition, an elewise layer was used to perform the elementwise multiplication between $G_{t-1}$ and the Gaussian weight mapping to generate ${\bar{G}}_{t-1}$. It reflected the complementary role of the different levels of response and helped to obtain better targeting capabilities.

Through this architecture, not only were the parameters of depth feature network $f^{l}$ trained but also the elementwise weight mapping and the regularization parameters $\lambda_{m}$ and $\lambda_{n}$ of RLR were learned. In short, the goal of the dynamic Siamese network architecture proposed in this paper was to realize online updatable closed-loop detection model training, not just fitting a detection function.

## 3. Closed-Loop Detection Training Considering the Spatiotemporal Correlation

In order to obtain rich spatiotemporal information of the mobile robot, the ability of the Siamese network to train learning on labeled video sequences was used [28]. All parameters involved in the real-time closed-loop detection model based on the dynamic Siamese network were learned offline. Therefore, during the operation of the robot, video sequence $\left\{S_{i}|i=1,\ldots ,I\right\}$ was obtained containing I-frame images to determine the target template at the closed-loop initial position through the model architecture as defined in Figure 4 and then obtain the response diagram $\left\{S_{i}|i=1,\ldots ,I\right\}$ of each frame in the video sequence. At the same time, for the *I*-frame sequence image, there were ground live images $\left\{J_{i}|i=1,\ldots ,I\right\}$ with the same size and one-to-one corresponding with $S_{i}$ where label 1 was used to represent the key frame candidate and label −1 was used to represent the background of the current frame. In order to solve the loss of each frame, the logistic loss function was defined as follows:

(9) $$Loss_{i}=\frac{1}{\left|S_{i}\right|}\log\left(1+\exp\left(-S_{i}⊙J_{i}\right)\right)$$

(10) $$Loss_{all}=\sum_{i=1}^{I}L_{i}$$

where $\left|S_{t}\right|$ represents the absolute value of $S_{t}$ and $Loss_{all}$ is the total loss of the whole video sequence. Through the back-propagation mechanism, the loss propagated to all parameters of the dynamic Siamese network including the elementwise weight mapping, two RLR layers, and relevant regularization parameters $\lambda_{m}$ and $\lambda_{n}$.

Compared with other closed-loop detection methods based on target detection, the proposed dynamic Siamese network integrated two new network layers, namely, RLR and CirConv. In order to make the closed-loop detection network model have a back-propagation mechanism and trainability of the random gradient descent, it was necessary to obtain the gradient of $L_{i}$ according to these two new network layers. As shown in Figure 4, given $\nabla_{\tilde{O}}Loss_{i}$, $\nabla_{F}Loss_{i}$, $\nabla_{F_{1}}Loss_{i}$, and $\nabla_{F_{1}}Loss_{i}$ were calculated through the CirConv and RLR network layers on the right to ensure that the loss was effectively propagated to $f^{l}$. Therefore, Equation (3) was defined to give priority to ensuring that $\nabla_{\tilde{O}}Loss_{i}$ was propagated to $\nabla_{M}Loss_{i}$:

(11) $$\nabla_{M}Loss_{i}=\Gamma^{-1}({\hat{F}}_{1}⊙{\hat{\nabla }}_{\tilde{O}}Loss_{i})$$

where ^ represents the Fourier transformation. $\nabla_{F}Loss_{i}$ and $\nabla_{\lambda_{v}}Loss_{i}$ were calculated based on Equation (4).

(12) $$\nabla_{F}Loss_{i}=\Gamma^{-1}(V⊙{\hat{\bar{F}}}_{1}⊙{\hat{\nabla }}_{V}Loss_{i})$$

(13) $$\nabla_{\lambda_{m}}Loss_{i}=\Gamma^{-1}{\left(-V^{2}⊙{\hat{\bar{F}}}_{1}⊙\hat{F}\right)}^{T}\nabla_{M}Loss_{i}$$

where $V={\left({\hat{\bar{F}}}_{1}⊙{\hat{F}}_{1}+\lambda_{m}\right)}^{-1}$, $\nabla_{F_{1}}Loss_{i}$ was calculated through $\nabla_{M}Loss_{i}$ and $\nabla_{\tilde{O}}Loss_{i}$.

(14) $$\nabla_{F_{1}}Loss_{i}=\Gamma^{-1}(M⊙{\hat{\nabla }}_{\tilde{O}}Loss_{i})+ℜ{\left(-2V^{2}⊙{\left({\hat{\bar{F}}}_{1}\right)}^{2}⊙\hat{F}\right)}^{T}{ℜ}^{H}\nabla_{M}Loss_{i}$$

where ℜ is the discrete Fourier transform matrix. The above solution process could also be used to calculate $\nabla_{F_{Z}}Loss_{i}$, $\nabla_{F_{G}}Loss_{i}$, $\nabla_{F_{\bar{G}}}Loss_{i}$, and $\nabla_{\lambda_{n}}Loss_{i}$. For the elementwise multi-layer fusion in Equation (8), $\Phi^{l}$ can be calculated by the following formula:

(15) $$\nabla_{\Phi^{l}}Loss_{i}=S_{i}^{l}⊙\nabla_{S_{i}}Loss_{i}.$$

## 4. Experimental Results and Analysis

In order to verify the performance of the vSLAM real-time closed-loop detection method based on the dynamic Siamese network, three datasets of Gardens Point, Nordland, and Mapillary were used for the experimental analysis. The accuracy of this method was compared with the closed-loop detection method based on BoVW, PlaceCNN, GiST, SS_PlaceCNN(Sliding window-based PlaceCNN) and AutoEncoder. The accuracy indicators included an accuracy recall rate and an average quasi removal rate. The computer software and the hardware environment of the experiment are shown in Table 1.

The closed-loop detection results were divided into four categories according to the algorithm prediction and facts, as shown in Table 2.

In order to intuitively measure the accuracy of the different closed-loop detection algorithms, the occurrence times of true positive (*TP*), false positive (*FP*), false negative (*FN*), and true negative (*TN*) were counted on a dataset, and the corresponding accuracy rate (*P*) and recall rate (*R*) were calculated. The specific calculation formula is as follows.

(16) P=TP/(TP+FP)

(17) R=TP/(TP+FN).

In Equation (16), *P* represents the proportion of the correct closed-loops among all closed-loops detected by the closed-loop detection algorithm. In Equation (17), *R* represents the probability that all actual closed-loops can be accurately detected by the closed-loop detection algorithm.

### 4.1. Dataset Analysis

For the analysis of the Gardens Point, Nordland and Mapillary datasets, see Table 3. These are classic test datasets of a closed-loop detection algorithm including three complex scenes: only illumination change, simultaneous illumination and viewing angle change, and simultaneous illumination and season change. They all retain spatiotemporal data information completely, which is very conducive to the offline parameter learning of the method proposed in this paper. Most traditional detection algorithms often ignore this rich information.

### 4.2. Design of the Experiment

Experiment 1: The robustness of the proposed method and the closed-loop detection algorithm based on BoVW, PlaceCNN, GiST, SS_PlaceCNN and AutoEncoder were compared for three complex scenes: only illumination change, simultaneous illumination and viewing angle change, and simultaneous illumination and season change.

Experiment 2: The average accuracy of various closed-loop detection algorithms was compared in the Gardens Point, Nordland, and Mapillary datasets.

Experiment 3: The time performance of the closed-loop detection algorithm was compared to explore the effectiveness of real-time closed-loop detection based on a joint training strategy. The Nordland dataset was selected as the experimental dataset.

### 4.3. Experimental Results and Analysis

The following subheadings provide a detailed analysis of the results of the three experiments designed in Section 4.2. The superiority and feasibility of the proposed method were demonstrated from the different perspectives, respectively.

#### 4.3.1. Robustness Analysis and a Comparison of the Closed-Loop Detection Algorithms

**Figure 5:**
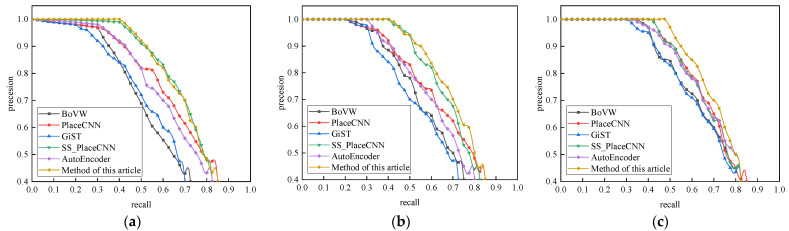
Comparison of the robustness of different closed-loop detection algorithms: (**a**) Gardens Point; (**b**) Mapillary; (**c**) Nordland.

Experiment 1: Figure 5a–c shows the robustness comparison between various closed-loop detection algorithms and the proposed method under different datasets containing complex scene changes. The overall analysis showed that the accuracy of the closed-loop detection algorithms decreased rapidly due to the influence of complex environment changes. The comparison results of the GiST and BoVW closed-loop detection algorithms under different datasets are at the bottom, which shows that the traditional closed-loop detection algorithm was easily affected by the change of illumination and shooting angle and the robustness was relatively poor. As can be seen from Figure 5b, when there was only an illumination change, the robustness of the closed-loop detection algorithm based on SS_PlaceCNN was close to that of the method in this paper and the performance was significantly higher than that of the detection algorithm using AutoEncoder. It shows that the method proposed in this paper could learn and convert the target appearance change and background suppression during the movement of mobile robot. The selection of the closed-loop key frames in different lighting scenes could be considered in time so that the detection algorithm was less affected by appearance changes.

Through comparative experiments on the three typical closed-loop detection datasets, the overall performance of the vSLAM real-time closed-loop detection method based on the dynamic Siamese network proposed in this paper was better than the other classical closed-loop detection algorithms. For scenes where only the illumination changed, the illumination and viewing angle changed at the same time, and the illumination and season changed at the same time, it ensured a high accuracy and good recall rate. It ensured a high accuracy when the recall rate was 75%.

#### 4.3.2. Analysis and Comparison of the Average Accuracy of the Closed-Loop Detection Algorithms

**Figure 6:**
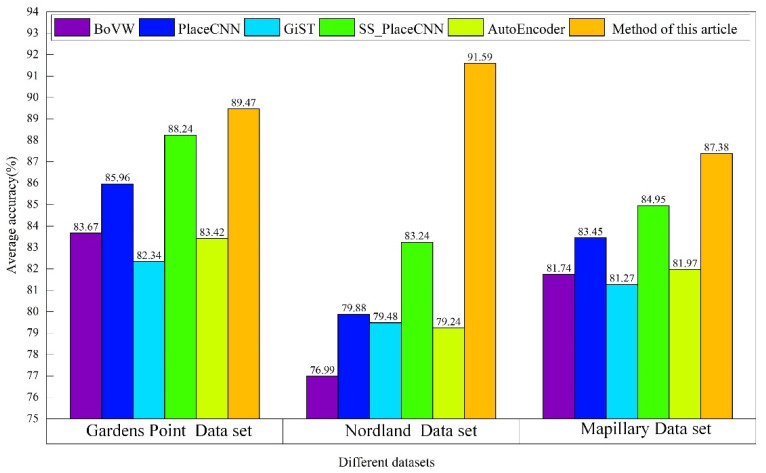
Average accuracy of the different algorithms with the different datasets.

Experiment 2: The average accuracy of each closed-loop detection algorithm under the three datasets of Gardens Point, Nordland, and Mapillary is shown in Figure 6. The environment of the Gardens Point dataset contained significant illumination changes and a certain degree of viewing angle changes. As the Nordland dataset was a long-distance video shot in different seasons and contained spatiotemporal data, the subsets in the different seasons showed the greatest change compared with the appearance. The Mapillary dataset environment contained appearance changes caused by moderate light changes (weather conditions and shooting time).

By analyzing Figure 6, it can be seen that the average accuracy of the vSLAM real-time closed-loop detection method based on the dynamic Siamese network proposed in this paper was higher than that of the other algorithms under the three scene datasets with different complex angles and reached the best in the Nordland dataset. The main reason was that the target appearance change and background suppression conversion mechanism proposed in this paper could effectively deal with the influence of exterior changes on key frame judgments during mobile robot movements. By integrating the multi-layer depth features, the proposed elementwise fusion strategy effectively ensured the accurate positioning of the key frames in the process of the closed-loop judgment and solved the problem of insufficient robustness of the key frame image selection in the closed-loop detection.

#### 4.3.3. Analysis and Comparison of the Time Performance of the Closed-Loop Detection Algorithms

Experiment 3: In order to verify the time performance of the real-time closed-loop detection algorithm proposed in this paper, the key frame accurate positioning time based on the dynamic Siamese network was compared with the other closed-loop detection algorithms. The key frame accurate positioning time was calculated by comparing the similarity in 1200 database images after the algorithm extracted the complete image features. The experimental results are shown in Table 4.

It can be concluded from Table 4 that the closed-loop detection speed of the method in this paper was the best, which effectively reduced the image feature extraction time in the closed-loop detection link. The joint training strategy designed in this paper could adapt to the offline parameter training considering the time–space correlation during the simultaneous positioning and map construction of the mobile robot, which could provide accurate optimization for the parameters of the closed-loop detection model, effectively solving the problem of insufficient real-time positioning of the key frame image in the closed-loop detection.

## 5. Conclusions

In this paper, a real-time closed-loop vSLAM detection method based on a dynamic Siamese network was proposed. Through a fast conversion learning model, it effectively captured the changes of target appearance and background suppression in the process of online learning robot mapping. An adaptive fusion strategy based on an elementwise strategy was proposed to further improve the positioning accuracy of key frames in the process of the closed-loop judgment. At the same time, the designed joint training strategy ensured that the constructed dynamic Siamese network could directly carry out offline training on the marked video sequence and make full use of the space–time information of the moving robot. The experimental results showed that this method had a higher robustness than the existing methods and could better achieve the balance of key frame positioning accuracy and real-time in complex scenes with changing illumination and viewing angles.

At present, it is still novel to use deep learning methods to solve the loop detection problem of vSLAM from different angles. In the future, with the rapid development of 5G networks and intelligent edge computing technology, the efficiency of this method in large-scale complex scenes can be further improved and a more ideal perception and application ability can be realized.

## Figures and Tables

**Table 1:** Introduction to the experimental environment.

Number	Type		Detailed Configuration
1	Hardware resource configuration	CPU	Intel(R)Xeon(R)Gold5118 CPU @ 2.30 GHz
2		GPU	NVIDIA Quadro P5000
3		Memory	Physical memory 128 G, dedicated GPU memory 16 g
4	Main software running environment and dependent environment	sys. platform	win32
5		Python	3.7.6 (MSC v.1916 64 bit (AMD64))
6		NumPy	1.18.1
7		detectron2	0.1.1
8		CUDA	10.1
9		PyTorch	1.4.0
10		GPU 0	Quadro P5000
11		Pillow	7.0.0
12		torchvision	0.5.0
13		cv2	4.2.0

**Table 2:** Classification of the closed-loop test results.

Facts/Forecasts	Closed-Loop	Non-Closed-Loop
Closed-loop	True positive	False negative
Non-closed-loop	False positive	True negative

**Table 3:** Dataset analysis.

Dataset	Introduction to the Dataset	Dataset Characteristics
Gardens Point	The dataset was collected on the campus of the University of Queensland (QUT), including three subdatasets in two days and one night.	The dataset has two characteristics: viewing angle change and illumination change. Each subdataset has 200 images.
Mapillary	It has 25 K street view images (18 K train, 2 K Val, 5 K test) with a wide resolution. The dataset is densely annotated (covering 98% of pixels) and contains 28 stuff and 37 thing categories.	(1) Changes in weather conditions (sun, rain, snow, fog, haze) and photographing time (dawn, day, dusk, night); (2) A wide range of camera sensors, different focal lengths, image aspect ratios, and different types of camera noise; (3) Different camera angles (road, sidewalk, and off road).
Nordland	A documentary about the Nordland railway produced by NRK, a railway line connecting Trondheim and Bode city.	By erecting cameras at the front of the train, 729 km-long railway lines are photographed in different seasons of spring, summer, autumn, and winter. The length of each video is about 10 h.

**Table 4:** Time performance analysis of the closed-loop detection.

Algorithm	BoVW	PlaceCNN	GiST	SS_PlaceCNN	AutoEncoder	Method Proposed
Dimension	600	1000	1500	8614	256	512
Time/s	17.65	19.47	20.16	61.58	23.26	12.28

## Data Availability

Data are contained within the article.
